# Neutrophil priming as a risk factor for surgical site infection in patients with colon cancer treated by laparoscopic surgery

**DOI:** 10.1186/s12893-019-0674-6

**Published:** 2020-01-06

**Authors:** Yuji Toiyama, Yoshinaga Okugawa, Tadanobu Shimura, Shozo Ide, Hiromi Yasuda, Hiroyuki Fujikawa, Yoshiki Okita, Takeshi Yokoe, Junichiro Hiro, Masaki Ohi, Masato Kusunoki

**Affiliations:** Department of Gastrointestinal and Pediatric Surgery, Division of Reparative Medicine, Institute of Life Sciences, Graduate School of Medicine, Mie University, Tsu, Mie 514-8507 Japan

**Keywords:** Neutrophil, Surgical site infection, Anastomotic leakage, Colon Cancer

## Abstract

**Background:**

The purpose of this study is to identify perioperative marker predicting postoperative surgical site infection (SSI) including with anastomotic leakage (AL) in curative colon cancer patients, laparoscopically.

**Methods:**

In total, 135 colon cancer patients (stage I–III) undergoing curative laparoscopic surgery between January 2004 and December 2013 were enrolled in this study. We collected data on clinicopathological factors, laboratory data on pre and postoperative day 3 (POD3) and tumor markers levels to assess the relation to surgical site infection (SSI) including with anastomotic leakage (AL).

**Results:**

SSI and AL occurred in 16 cases (5.6%) and 4 cases (3%), respectively. SSI and AL were not association with clinicopathological factors. Within laboratory data and tumor markers preoperatively, high neutrophil counts were significantly associated with SSI (*P* < 0.05) and AL (*P* < 0.01), respectively. Area under curves (AUC) of SSI and AL were 0.656 and 0.854, respectively. In addition, high neutrophil counts on POD3 also were significantly associated with SSI (*P* < 0.01) and AL (*P* < 0.01), respectively. Area under curves (AUC) of SSI and AL were 0.747 and 0.832, respectively.

**Conclusion:**

Neutrophil count on pre and POD3 are potentially valuable indicators of SSI including with AL in colon cancer patients undergoing curative surgery laparoscopically.

## Background

Surgical site infection (SSI) is the most common complication after digestive tract surgery, despite advances in surgical techniques [1]. In particular, elective surgery in the field of colon and rectum is associated with a high rate of SSI, [2] which clearly prolongs length of hospital stay and increases costs of medical care [3].

The Centers for Disease Control and Prevention (CDC)’s National Nosocomial Infections Surveillance (NNIS) system made a proposal of risk index that scored each procedure by counting the number of risk factors that included an American Society of Anesthesiologists (ASA)-performance status more than 3, an operation classified as contaminated or dirty-infected, and prolonged operative duration [4]. Although this risk index is widely used and comprehensive, it is not specific for organs, diseases, or surgical procedures. In addition, postoperative infection complications (PICs), including SSI and remote infection, after colorectal surgery is known to be associated with preoperative several factors such as age, sex, poor nutrition, prior surgery, comorbidities, obesity, and malignant disease [5].

Laparoscopic surgery greatly reduces the risk of SSI compared with open surgery [6]. Imai et al. demonstrated that open operative procedures in colon cancer cases to be a risk factor for SSI, whereas laparoscopic surgery was associated with a significantly lower risk of SSI [7]. Therefore, independent evaluation of SSI risk factors in patients with colon cancer who undergo laparoscopic surgery is important.

In this study, we investigated factors that might facilitate early diagnosis of SSI and AL after laparoscopic colon cancer surgery.

## Methods

### Patients

We retrospectively enrolled 135 patients with curative laparoscopic surgery for colon cancer (stages I–III) at the Department of Gastrointestinal and Pediatric Surgery in Mie University hospital from January 2004 to December 2013. The tumor-node-metastasis (TNM) staging system from the American Joint Committee on Cancer was used for the pathological tumor staging of CRC. Curative resection was defined as macroscopically and microscopically undetectable tumors. No neoadjuvant chemotherapy or radiotherapy was given to all patients in this study, and no mortality in the perioperative period was observed.

SSI definitions include superficial and deep incisional infections, and organ/ space infections, that were classified based on the criteria of the CDC [8]. Organ/space SSI included AL or intra-abdominal abscesses characterized by the following: The presence of septic fluid in the peritoneal space demonstrated by surgical drainage or needle aspiration and established bacteriologic culture. Postoperative AL was diagnosed by performing colonic enemas. In contrast, incisional SSI included wound infections characterized by the following: The presence of purulent fluid or pus in the wound incision, and local redness and warming at the surgical site.

All patients received second-generation cephalosporin antibiotics (cefmetazole) for prophylaxis, which was continued for 1 day after surgery according to the 1999 guidelines for prevention of SSI [8].

We analyzed the associations between SSI and AL, and clinicopathological factors; operative factors (operation time, blood loss) and changes between presurgical and POD3 laboratory data. Blood collection and subsequent analyses were approved by the Institutional Review Boards of Mie University Hospital in Japan. Written informed consent was obtained from all patients. The study was conducted in accordance with the guidelines of the 1996 Declaration of Helsinki.

### Statistical analysis

All statistical analyses were carried out using Medcalc 7.2 for Windows (Broekstraat 52, 9030, Mariakerke, Belgium). Quantitative data are presented as means ± standard deviation (SD) or means ± standard error (SE). Data were compared using the Mann–Whitney, Kruskal–Wallis test, or the χ^2^ test, as appropriate. Receiver operating characteristic (ROC) analysis was performed to determine the diagnostic performance of several factors for distinguishing colon cancer patients with SSI or AL from those without SSI or AL. Area under the curve (AUC) values reflect the probability of correctly identifying colon cancer patients with SSI or AL from those without. Optimal cutoff thresholds for diagnosis were obtained by Youden’s index. All *P-*values were two-sided. *P* < 0.05 was considered significant.

## Results

### Patient characteristics

The study group included 66 men and 69 women, with a median age of 69 years (range: 43–87 years). Of the 135 registered colon cancer patients, 9 (6.6%) had tumors located in the cecum, 66 (48.8%) in ascending colon, 10 (7.4%) in transverse colon, 14 (10.3%) in descending colon, and 36 (26.6%) in sigmoid colon. Twelve patients (8.8%) had stage 0 disease, 35 (28.8%) stage I, 47 (34.8%) stage II, and 37 (27.4%) stage III disease. Their median tumor size was 30 mm (range: 3–85 mm); median surgical time was 213 min (range:101–492 min); median operative blood loss was 35 ml (range: 0–406 ml); and median body mass index (BMI) was 23.2 (range: 15.8–31.1).

### Association between clinicopathological findings and postoperative SSI and AL

SSI occurred in 16 (11.8%) and AL occurred in 4 patients (2.9%; Table 1). Patients who suffered SSI, or AL, did not significantly differ from other patients with regard to age, sex, ASA classification, pathological findings, operative factors, tumor location or BMI.

**Table 1:** Associations between clinicopathological findings and postoperative SSI and AL

Category			SSI (−)	SSI (+)	*p*-Value	AL (−)	AL (+)	*p*-Value
			(119/135)	11.8% (16/135)		(131/134)	3% (4/135)	
Age	≦69 y	*N* = 69	60	9	NS	67	2	NS
	>69 y	*N* = 66	59	7		64	2	
Gender	Male	N = 66	61	5	NS	65	0	NS
	Female	N = 69	58	11		65	4	
ASA classification	1	*N* = 40	35	5	NS	39	1	NS
	2	*N* = 60	52	8		58	2	
	3	*N* = 34	30	4		33	1	
Tumor size	≦30 mm	*N* = 81	71	10	NS	80	1	NS
	>30 mm	*N* = 52	46	6		49	3	
Pathological T stage	T1–2	*N* = 70	64	6	NS	69	1	NS
	T3–4	*N* = 65	55	10		62	3	
Pathological N stage	N-	*N* = 98	87	11	NS	96	2	NS
	N+	*N* = 37	32	5		35	2	
Operative time	Mean (SD)	(min)	226 (69)	232 (57)	NS	227 (68)	228 (46)	NS
Blood loss	Mean (SD)	(mL)	53 (65)	61 (61)	NS	54 (65)	36 (15)	NS
Tumor location	Cecum	N = 9	6	3	NS	9	0	NS
	Ascending	N = 66	59	7		64	2	
	Transverse	*N* = 10	10	0		10	0	
	Descending	*N* = 14	12	2		14	0	
	Sigmoid	*N* = 36	32	4		34	2	

*AL* Anastomotic leakage, *ASA* The American Society of Anesthesiologists, *BMI* Body mass index, *NS* Not significant, *SD* Standard deviation, *SSI* Surgical site infection

### High neutrophil counts predict SSI and AL

**Fig. 1:**
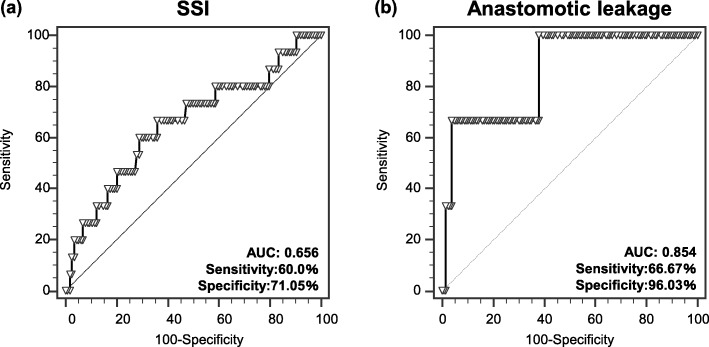
Receiver operator curves show the accuracy of preoperative neutrophil counts in predicting (**a**) surgical site infection (SSI) and (**b**) anastomotic leakage (AL)

Table 2 shows the results of association between preoperative laboratory factors and postoperative infectious complication. Preoperative serum CRP and albumin levels were not significant between SSI negative and positive. In addition, preoperative tumor markers [**Carcinoembryonic antigen** (CEA) and carbohydrate antigen (CA) 19–9] also do not differentiate between SSI positive and SSI negative. In contrast, within preoperative hematocyte, neutrophil counts in colon cancer patients with SSI positive are significantly higher than that in SSI negative (*p* < 0.05). Similarly, neutrophil counts in colon cancer patients with AL positive are also significantly higher than that with AL negative (p < 0.05), however, other preoperative laboratory factors we investigated showed no difference between AL positive and negative. Receiver operative curve (ROC) revealed that AUCs of preoperative neutrophil counts for prediction of postoperative SSI and AL are 0.656 (sensitivity: 60.0%, specificity: 71.05%, cut-off value: 3990) and 0.854 (sensitivity: 66.67%, specificity: 96.03%, cut-off value: 5300), respectively (Fig. 1a and b).

**Table 2:** Associations between preoperative laboratory factors and postoperative infectious complications

Blood factors (Preoperative)	SSI (−)	SSI (+)	*p*-Value	AL (−)	AL (+)	*p*-Value
	mean (SD)	mean (SD)		mean (SD)	mean (SD)	
Neutrophil counts (/uL)	3501 (1499)	4373 (2020)	<0.05	3534 (1477)	6471 (3406)	<0.01
Lymphocyte counts (/uL)	1620 (553)	1582 (529)	NS	1620 (550)	1410 (546)	NS
CRP (mg/dL)	0.19 (0.40)	0.17 (0.24)	NS	0.19 (0.39)	0.07 (0.05)	NS
Alb (g/dL)	4.2 (0.40)	4.1 (0.32)	NS	4.2 (0.39)	4.2 (0.30)	NS
CEA (ng/mL)	5.9 (7.2)	8.2 (11.5)	NS	5.9 (7.3)	22.9 (19.8)	NS
CA19–9 (U/mL)	18.4 (15.1)	24.6 (24.9)	NS	19.1 (16.6)	19.0 (7.0)	NS

*AL* Anastomotic leakage, *Alb* Albumin, *CA19–9* Carbohydrate antigen 19–9, *CEA* Carcinoembryonic antigen, *CRP* C-reactive protein, *NS* Not significant, *SD* Standard deviation, *SSI* Surgical site infection

### High neutrophil counts on postoperative day 3 predict SSI and AL

Table 3 shows the relationships between POD3 laboratory factors and SSI and AL. Colon cancer patients with high serum CRP and those with low albumin levels were significant more likely to suffer SSI occurrence (CRP: *P* < 0.05; Albumin: *P* < 0.01). In addition, POD3 neutrophil counts in colon cancer patients with SSI were significantly higher than in patients who did not develop SSI (*P* < 0.01). Similarly, neutrophil counts in patients with AL were significantly higher than in patients who did not develop AL (*P* < 0.05); however, other preoperative laboratory factors we investigated did not significantly differ between patients who did and did not suffer AL. The AUCs of POD3 neutrophil counts in predicting SSI were 0.747 (sensitivity: 81.25%, specificity: 64.81%, cut-off value: 5290) and 0.832 for AL (sensitivity: 100%, specificity: 65.83%, cut-off value: 5720; Fig. 2a, b).

**Table 3:** Associations between laboratory factors on POD3 and SSI and AL

Blood factors (POD3)	SSI (−)	SSI (+)	*p*-Value	AL (−)	AL (+)	*p*-Value
	mean (SD)	mean (SD)		mean (SD)	mean (SD)	
Neutrophil counts (/uL)	5230 (2088)	7188 (2369)	<0.01	5398 (2192)	7735 (1588)	<0.01
Lymphocyte counts (/uL)	1118 (438)	947 (215)	NS	1097 (426)	1062 (102)	NS
CRP (mg/dL)	9.0 (5.7)	13.3 (6.8)	<0.05	9.5 (5.9)	12.8 (7.4)	NS
Alb (g/dL)	3.4 (0.33)	3.2 (0.23)	<0.01	3.4 (0.33)	3.1 (0.25)	NS

*AL* Anastomotic leakage, *Alb* Albumin, *CRP* C-reactive protein, *NS* Not significant, *POD* Postoperative day, *SD*: standard deviation, *SSI* Surgical site infection

**Fig. 2:**
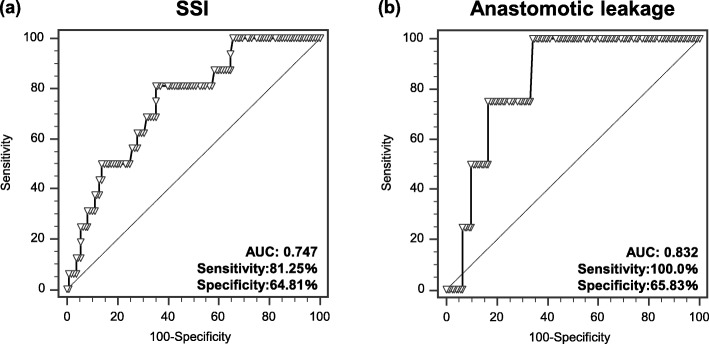
Receiver operator curves show the accuracy of neutrophil counts on post-operative day 3(POD3) in predicting (**a**) surgical site infection (SSI) and (**b**) anastomotic leakage (AL)

**Fig. 3:**
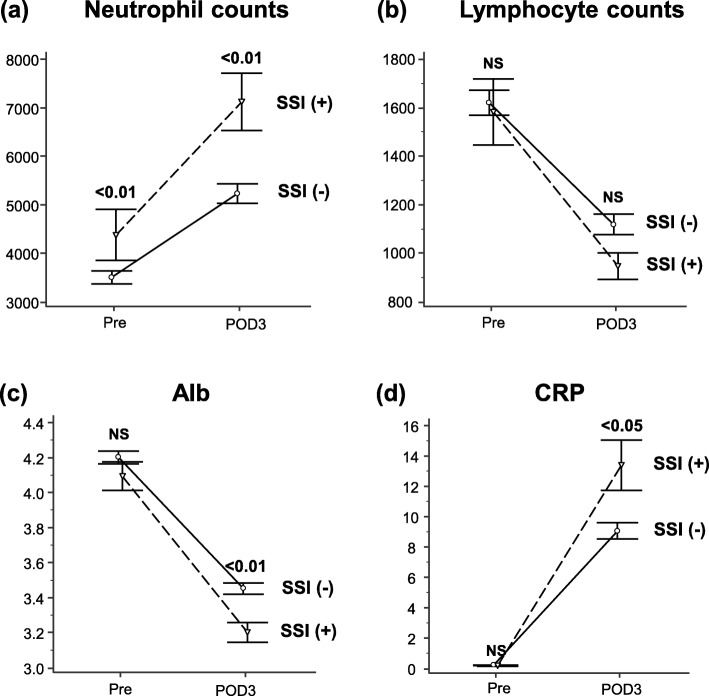
Serial changes in serum markers by surgical site infection (SSI) status. (**a**) Neutrophil count, (**b**) lymphocyte count (**c**) albumin (Alb) concentration, and (**d**) C-reactive protein (CRP)

**Fig. 4:**
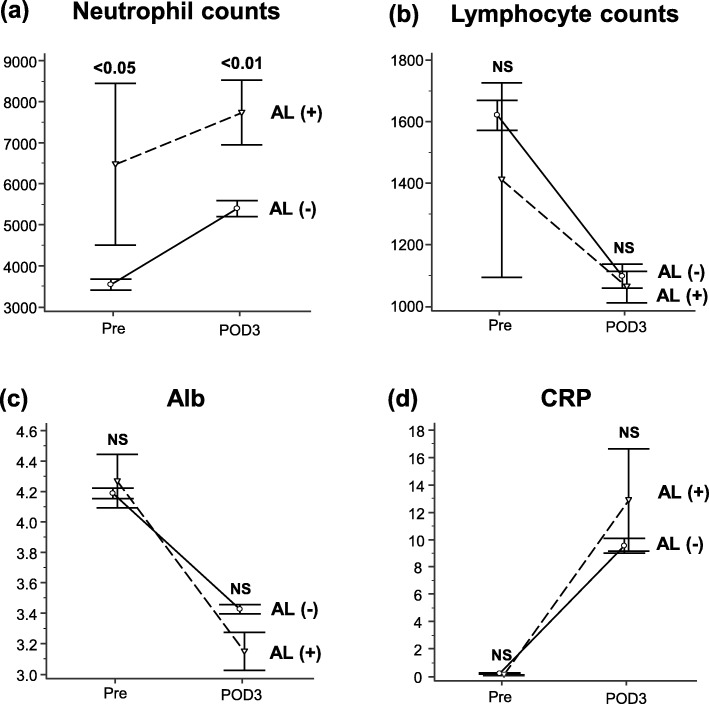
Serial changes in serum markers by postoperative anastomotic leakage (AL) status. (**a**) Neutrophil count, (**b**) lymphocyte count, (**c**) albumin (Alb) concentration, and (**d**) C-reactive protein (CRP)

We then investigated changes between presurgical and POD3 laboratory data. Interestingly, only neutrophil counts stayed significantly higher in patients with SSI **(**Fig. 3a-d**)** and AL (Fig. 4a-d**)**.

## Discussion

Current study showed that presurgical and POD3 high neutrophil counts can help distinguish colon cancer patients after curative laparoscopies with SSIs and AL from those without SSIs and AL (Cut-off values of presurgical neutrophil count for SSIs and AL are 3990 and 5300, respectively; Cut-off values of POD3 neutrophil count for SSIs and AL are 5290 and 5720, respectively).

Several studies have shown that preoperative neutrophil activation may be a risk factor for PICs [9–11]. We previously demonstrated that preoperative increased levels of both neutrophil counts and neutrophil elastase were significantly associated with occurrence of SSI and AL in patients with ulcerative colitis [9]. In addition, ulcerative colitis patients with high preoperative levels of neutrophil elastase showed even higher levels of neutrophil elastase postoperatively, and consequently, SSIs [9]. Furthermore, perioperative application of leukocyte apheresis, which removes activated leukocytes from blood circulation, can control neutrophil hyper activation, leading to reduction of postoperative SSIs in patients with ulcerative colitis [10, 11]. These evidences show that perioperative neutrophil activation might be a surgery-dependent risk factor for septic complications.

Neutrophils are the most important effector cells of the innate immune system against invasion of microbial cells. However, excessive activation of neutrophils at inappropriate sites leads to disruption of normal tissues in host [12, 13]. Therefore, appropriate migration of neutrophils into surgical sites is extremely important to prevent SSI. First of all, neutrophils in circulation move to postcapillary venules surrounding local sites with infection or injury. Then, neutrophils firmly attach to the vascular endothelium, and finally, neutrophils migrate into the extravascular inflammatory sites to phagocytose the pathogen such as bacteria [14]. Collectively, derangement of the sequential steps of neutrophil recruitment into local inflammatory sites may be an important determinant of PICs. Previous report, using a small cohort of patients with gastrointestinal cancer (*n* = 31), showed that preoperative neutrophil counts of patients who developed postoperative infections tended to be higher than that in the noninfected group, and high preoperative neutrophil adhesion capacity was closely associated with PICs [15]. In addition, we previously demonstrated that high preoperative neutrophil counts and long duration of operation are independent risk factors for o/sSSIs in patients with gastric cancer undergoing curative surgery [16]. Furthermore, In vivo study using solid tumor models showed that malignant tumors can increase peripheral blood neutrophils, that are sensitized toward neutrophil extracellular traps (NETs) formation [17]., and the study also demonstrated that, under these conditions, spontaneous microthrombosis associated with NET forms at the site of “second hit,” such as low-grade infection or surgery [17]. These results suggested that NET-induced microthrombosis lead to plugging of microvessels, deteriorated microcirculation and subsequent inappropriate immune responses at surgical sites, resulting in development of postoperative SSI [16].

Colorectal surgical procedures are associated with higher SSI incidence compared with other digestive tract. Previous report demonstrated that postoperative SSIs occur in 30–40% of the patients when prophylactic antibiotics are not administered [18]. Therefore, the CDC guidelines recommend that single-dose of an antibiotic is administered within 1 h before operation, and the administration period is within 24 h after the end of surgery [8, 19]. On the other hand, a questionnaire survey by the Japan Society for Surgical Infection revealed the duration of antibiotic administration within 3 days postoperatively accounting for 96% of cases [20]. This greatly differs from the US guideline. In our results, preoperative neutrophil priming among colon cancer patients treated with curative laparoscopies might be a strong predictor of SSI and AL, which implies that preoperative neutrophil counts could preoperatively indicate appropriate chemoprophylaxis administration periods.

Our second finding showed that neutrophil counts on POD3 were also significantly associated with SSI and AL. Even though clinical symptoms are not present, high postoperative neutrophil levels might help guide such decisions as whether to continue a starvation cure, to strengthen nutritional support and intensive anti-septic therapy, and to intervene surgically to prevent progression of potentially severe septic conditions.

We acknowledge several potential limitations in the present study. Generally, intraoperative factors such as operative time and blood transfusion are known to be risk factors for PICs, [21] along with several host factors, including age, comorbidities, and malnutrition [22, 23]. However, these recognized factors did not reach statistical significance in this study, probably because of the small sample size and the retrospective, single-institution nature of the study. In addition, the small sample size for complications that are quite unfrequent limits the clinical relevance of this study. However, the surgical procedures (R0 resections with D2- or D3-lymphadenectomy depending on clinical TNM stage), standard precautions for SSI, laboratory examinations, and follow-up were uniform throughout the entire study period.

## Conclusion

In conclusion, to our knowledge, this study is the first to show that after curative laparoscopies, colon cancer patients who developed SSIs presented significantly increased neutrophil counts, both presurgically and on POD3, compared with those without SSIs. Moreover, presurgical and POD3 high neutrophil counts can help distinguish such patients with AL from those without AL. This indicates that presurgical and POD3 neutrophil counts in colon cancer patients treated with curative laparoscopies should be regularly tracked.

## Data Availability

The datasets analyzed during the current study are available from the corresponding author on reasonable request.

## Abbreviations

AL: Anastomotic leakage
AUC: Area under curve
CA19–9: Carbohydrate antigen 19–9
CEA: Carcinoembryonic antigen
CRP: C-reactive protein
SSI: Surgical site infection
